# Arthroscopic-assisted uni-portal spine surgery via modified interlaminar approach combined with annular suturing for L5/S1 disc herniation: a case report and technical note

**DOI:** 10.3389/fsurg.2026.1807236

**Published:** 2026-04-20

**Authors:** Zhijun Chen, Wenrong Yang, Jincai Liu, Zhigang Zhao, Nan Zhou, Guangmin Pu, En Song

**Affiliations:** 1 Department of Orthopaedics, Chuxiong Yi Autonomous Prefecture Hospital of Traditional Chinese Medicine, Chuxiong, China; 2 Department of Sports Medicine, The First Affiliated Hospital of Kunming Medical University, Kunming, China

**Keywords:** annular suturing, arthroscopic-assisted uni-portal spine surgery, case report, L5/S1 lumbar disc herniation, modified interlaminar approach

## Abstract

**Background:**

Lumbar disc herniation (LDH) is a common clinical spinal disorder, with the L5/S1 segment being a frequently affected site due to its unique anatomical and biomechanical characteristics. Conventional minimally invasive spinal endoscopic techniques, such as percutaneous transforaminal endoscopic discectomy (PTED), percutaneous endoscopic interlaminar discectomy (PEID), and unilateral biportal endoscopy (UBE), have inherent limitations in treating L5/S1 LDH. These include difficulty bypassing the high iliac crest (for PTED), a steep learning curve (for PEID), and potential impairment of spinal stability (for UBE). To address these challenges, this study applied Arthroscopic-assisted uni-portal spine surgery (AUSS) via a modified interlaminar approach combined with 4-0 absorbable suture annular repair for L5/S1 LDH, reporting its short-term clinical outcomes and detailing key technical points.

**Case presentation:**

A 45-year-old male patient presented with a 1-year history of low back pain, which worsened over 1 month with persistent right lower limb radicular pain, unresponsive to conservative treatment. Preoperative lumbar MRI and CT confirmed L5/S1 disc herniation, with T2-weighted MRI showing low signal intensity of the herniated disc and axial CT demonstrating direct compression of the right S1 nerve root by the herniated nucleus pulposus. The patient underwent the modified procedure: during surgery, a portion of the ligamentum flavum was excised to expose the herniated nucleus pulposus, while the remainder was retracted and preserved. After complete removal of the herniated nucleus pulposus, full-thickness annular suturing was performed using 4-0 absorbable sutures, with knot tying performed extracanalicularly and pushed into place using a dedicated knot pusher. At 1, 3, and 12 months postoperatively, the incision healed well without complications such as infection, nerve injury, or cerebrospinal fluid leakage. Imaging re-evaluation showed no recurrence of L5/S1 disc herniation and a smooth posterior annular margin. The patient experienced significant relief of low back and leg pain, resuming normal daily activities within 1 month postoperatively. The visual analogue scale (VAS) score decreased from 7 preoperatively to 1, the Japanese Orthopaedic Association (JOA) score reached 25, and the Oswestry Disability Index (ODI) decreased from 68% preoperatively to 12%.

**Conclusion:**

Arthroscopic-assisted uni-portal spine surgery via the modified interlaminar approach combined with annular suturing is a safe, feasible, and effective treatment for L5/S1 LDH. Its core advantages include bypassing anatomical barriers such as the high iliac crest, maximizing the preservation of spinal osseous and ligamentous structures, ease of operation, high surgical efficiency, and a low short-term recurrence rate. This procedure is a targeted optimization of the traditional interlaminar approach, providing a valuable treatment option for L5/S1 LDH, especially in cases where conventional endoscopic techniques are limited. However, the results of this single-case study cannot be generalized to long-term efficacy, and large-sample, multi-center follow-up studies are needed for further validation.

## 1 Case presentation

### 1.1 General information

A 45-year-old male patient was admitted in December 2023 with a chief complaint of “recurrent low back pain for 1 year, aggravated with right lower limb radiating pain for 1 month”. One year prior, the patient developed dull pain in the lower back without obvious cause, which worsened with exertion and slightly relieved with rest, without regular treatment. One month before admission, the low back pain suddenly increased after bending over for labor, accompanied by persistent radiating pain from the right buttock, posterior thigh, and lateral calf to the dorsum of the foot, with numbness on the dorsum of the right foot, and no significant relief of symptoms when walking or lying flat. After 1 month of conservative treatment including bed rest, physical therapy, and oral non-steroidal anti-inflammatory analgesics outside the hospital, the symptoms did not improve, and gait instability and difficulty walking appeared, severely affecting quality of life and sleep, leading to consultation at our hospital.

**Figure 1:**
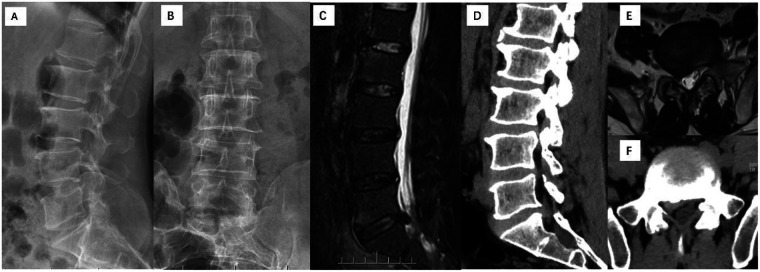
Preoperative imaging findings of the patient. **(A,B)** Preoperative anteroposterior and lateral radiographs. **(C)** Preoperative sagittal T2-weighted MRI. **(D)** Preoperative sagittal CT. **(E)** Preoperative axial T2-weighted MRI. **(F)** Preoperative axial CT.

Admission physical examination: The physiological curvature of the lumbar spine was straightened, with tenderness (+) at the L5/S1 spinous process space and right paraspinal area, right straight leg raising test 30° (+), strengthening test (+), right dorsiflexion muscle strength of the foot was grade 4, superficial sensation on the dorsum of the foot was decreased, bilateral knee and Achilles tendon reflexes were symmetrically present, and pathological signs were not elicited. Preoperative anteroposterior and lateral lumbar radiographs showed degenerative changes of the lumbar spine with slight narrowing of the L5/S1 intervertebral space; sagittal T2-weighted MRI showed posterior protrusion of the L5/S1 intervertebral disc with decreased signal intensity; axial MRI and CT showed compression of the right S1 nerve root by the herniated nucleus pulposus (Figure 1). Preoperative VAS score was 7, JOA score was 11, and ODI was 68%.

### 1.2 Surgical method

**Figure 2:**
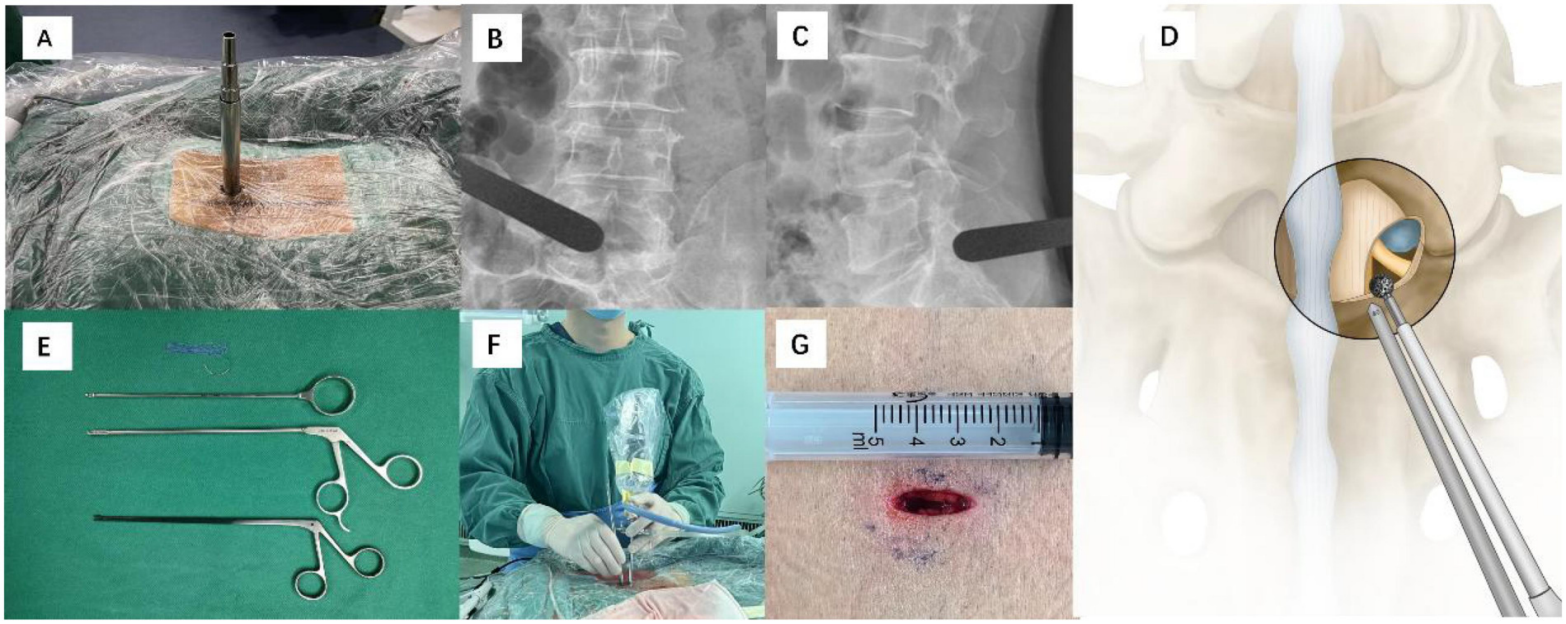
AUSS annular suturing device, intraoperative C-arm fluoroscopy positioning, and schematic illustration of via modified interlaminar approach. **(A)** Graduated dilators and surgical incision. **(B)** Anteroposterior fluoroscopic view showing dilators positioned at the tip of the right inferior articular process of L5. **(C)** Lateral fluoroscopic view showing dilators at the L5/S1 intervertebral space. **(D)** Schematic representation of the corner approach. **(E)** Annular suturing device components, from top to bottom: 4-0 absorbable suture needle, knot pusher, suture cutter, and 2 mm ultra-thin nucleus pulposus clamp. **(F)** AUSS handheld arthroscope and auxiliary handheld instrument. **(G)** Surgical incision.

The patient underwent general anesthesia and was placed in the prone position with the abdomen suspended. C-arm fluoroscopy was used to locate the L5/S1 segment, and the tip of the right inferior articular process of L5 was marked as the target for puncture and operation. A longitudinal skin incision of approximately 18 mm was made along the target. Graduated dilators were used to sequentially expand the subcutaneous tissue and paraspinal muscles. Under fluoroscopic guidance, the dilator was positioned at the tip of the right inferior articular process of L5 and the L5/S1 intervertebral space, and the working channel was inserted and fixed (Figures 2A–C). A 4 mm 30° arthroscopic system (Arthrex Synergy, USA) connected to a 4K imaging system was introduced, with continuous gravity irrigation of normal saline to maintain a clear surgical field. A radiofrequency ablation device (Backbone Biotechnology Co., Ltd., Anhui, China) was used for soft tissue dissection and hemostasis, clearly exposing the tip and medial margin of the right inferior articular process of L5 and the superior margin of the S1 lamina (Figure 2D). The arthroscope is held in the left hand for observation, while instruments are manipulated with the right hand (Figure 2F).

**Figure 3:**
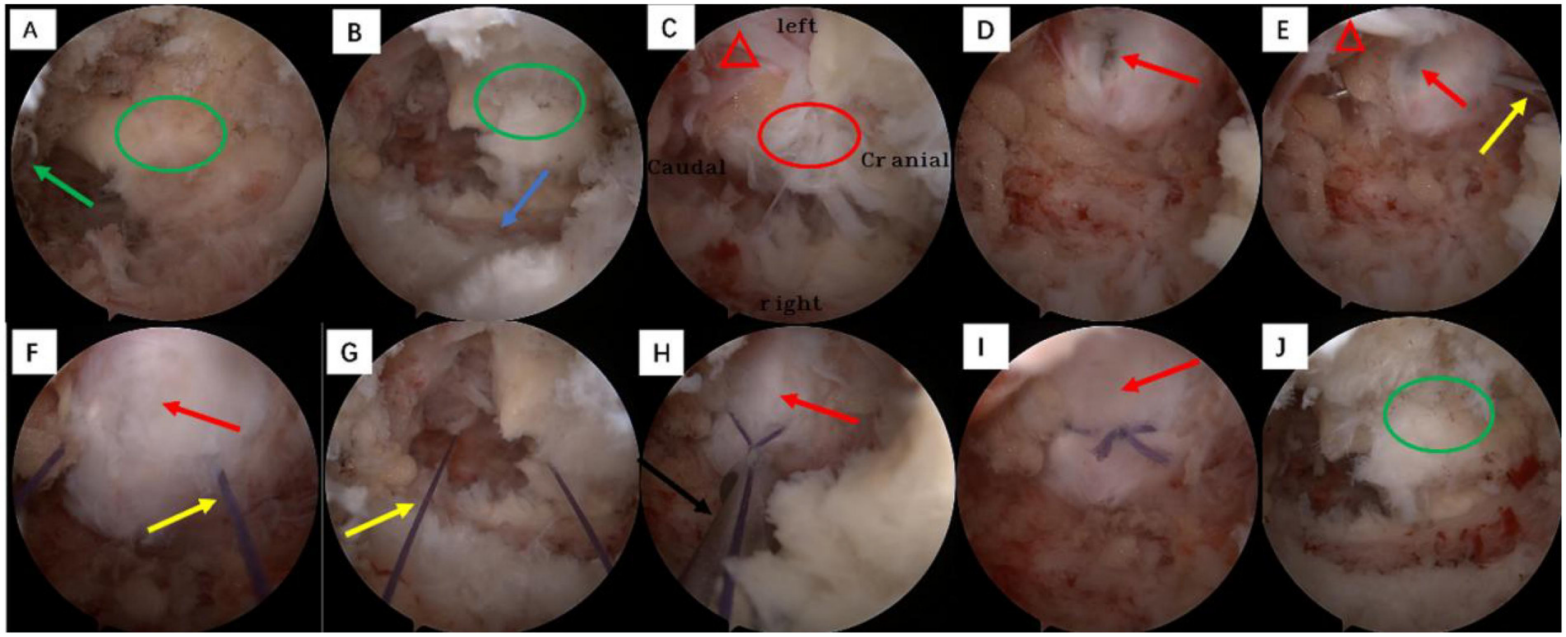
Application of the 4-0 absorbable suture annular fibrosus repair technique via modified interlaminar approach under AUSS. Endoscopic view orientation: 3 o'clock, cranial; 9 o'clock, caudal; 12 o'clock, left; 6 o'clock, right. **(A,B)** Removal of bony margins (green arrow) from the superior edge of the S1 lamina and the tip of the inferior articular process of L5 (blue arrow), exposing the caudal attachment of the ligamentum flavum (green ellipse). **(C)** Exposure of the herniated nucleus pulposus (red ellipse) and S1 nerve root (red triangle). **(D)** Annular fibrosus tear (red arrow) exposure following nucleus pulposus removal. **(E,F,G)** Suturing the annular tear using a 4-0 absorbable monofilament needle (yellow arrow), inserting approximately 2 mm caudal from the tear margin, and securing the annulus by knotting. **(H,I)** Knot pusher (black arrow) advanced to form a surgical knot, fully closing the annular fibrosus (red arrow) and trimming excess suture. **(J)** Final surgical view demonstrating preservation of most of the ligamentum flavum (green ellipse).

A 4.0 mm powered burr (Zirui Technology, Guizhou, China) and Kerrison rongeur were used to remove a small amount of bone from the superior margin of the S1 lamina to the caudal attachment of the ligamentum flavum, and a small amount of bone from the tip and medial side of the inferior articular process of L5. The ligamentum flavum was split along its longitudinal fibers, and a local area was excised to clearly expose the herniated nucleus pulposus, while the remaining ligamentum flavum was retracted medially with a nerve retractor and preserved throughout the procedure (Figures 3A, B). Radiofrequency hemostasis and surrounding tissue release were performed again to fully expose the dural sac, S1 nerve root, and herniated nucleus pulposus (Figure 3C).

The right S1 nerve root was gently retracted with a nerve retractor, and a 2 mm nucleus pulposus forceps was used to completely remove the herniated nucleus pulposus, avoiding enlargement of the original annular tear during operation; the annular defect was clearly visualized after nucleus pulposus removal (Figure 3D). Annular suturing requires 4-0 absorbable sutures, a knot pusher, a suture cutter, and a 2 mm ultra fine nucleus pulposus clamp (Figure 2E). A 2 mm ultra-thin nucleus pulposus clamp was used to hold a 4-0 absorbable suture needle (3L Medical Products Group Co., Ltd., Jiangxi, China). The needle was inserted approximately 2 mm from the posterior edge of the annular defect, passed full-thickness through the annular fibrosus, and exited approximately 2 mm anterior to the defect, with both ends of the suture drawn out to the subcutaneous tissue outside the spinal canal (Figures 3E–G).

A surgical knot was tied extracanalicularly, and a dedicated knot pusher (3L Medical Products Group Co., Ltd., Jiangxi, China) was used to slowly push the knot into the body, fitting it against the surface of the annular defect with appropriate tension to ensure tight closure of the annular defect and avoid contact of the knot with the nerve root. After a second reinforcing knot was tied, excess suture was cut with a suture clipper (Figures 3H, I). After endoscopic confirmation of adequate nerve root decompression, secure annular suturing, and no active bleeding, the endoscope and surgical instruments were removed, a drainage tube was placed in the incision, and the incision was closed layer by layer. The surgical incision is shown in Figure 2G.

### 1.3 Postoperative management and follow-up

The drainage tube was removed 24 h postoperatively, and the patient was instructed to perform straight leg raising exercises to prevent nerve root adhesion; on the second postoperative day, the patient was allowed to ambulate with a lumbar brace, avoiding bending, twisting, and weight-bearing. Postoperatively, routine symptomatic treatment including antibiotics for infection prevention, dehydration and detumescence, and neurotrophic therapy was administered for 3 days. The patient was discharged 1 week postoperatively and had suture removal at the outpatient clinic 2 weeks later.

Outpatient and imaging follow-ups were conducted at 1, 3, and 12 months postoperatively to record the patient's low back and leg pain symptoms, neurological function recovery, and to review lumbar CT and MRI to evaluate recurrence of disc herniation, annular healing, and preservation of spinal structures. Clinical efficacy was assessed using VAS, JOA, and ODI scores.

## 2 Results

The total operation time was approximately 45 min, with an estimated intraoperative blood loss of approximately 20 mL, and no intraoperative complications such as nerve root or dural sac injury. Postoperatively, the patient experienced immediate relief of right lower limb radicular pain and significant reduction in low back pain. At 1, 3, and 12 months postoperatively, the incision healed by primary intention without complications such as infection, cerebrospinal fluid leakage, nerve root adhesion, or lower limb muscle weakness; the patient's low back and leg pain completely resolved, gait returned to normal, and normal daily work and life were resumed without discomfort such as lower limb numbness or weakness. At 1 month postoperatively, the VAS score was 1, JOA score was 25, and ODI was 12%; these scores remained stable at 3 and 12 months postoperatively, with neurological function returning to normal levels.

**Figure 4:**
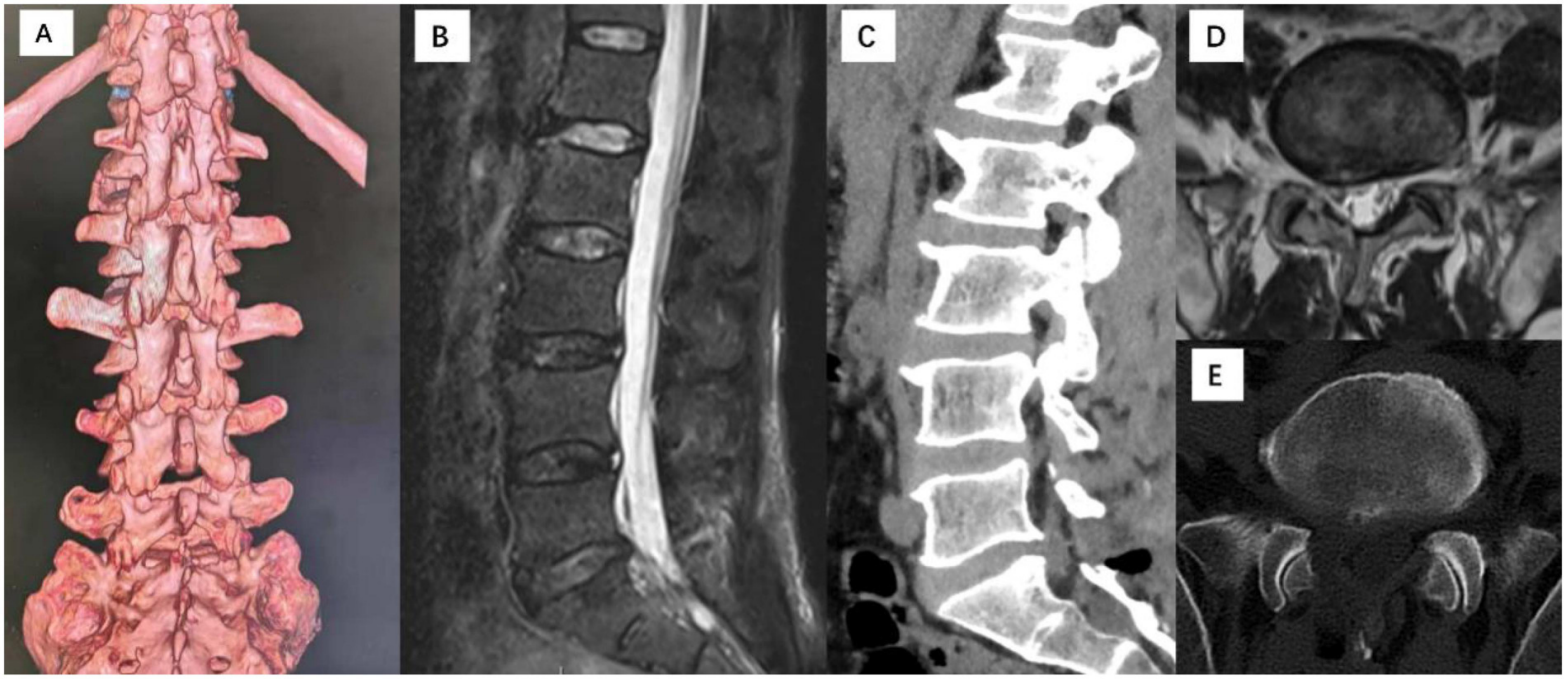
Postoperative imaging follow-up assessment. **(A)** Three-dimensional CT images demonstrate minimal bone removal from the superior edge of the right S1 lamina and the medial margin of the facet joint, with preservation of the bone at the inferior edge of the L5 lamina. **(B,C,D,E)** MRI and CT images confirm successful removal of the herniated nucleus pulposus and complete decompression of the spinal cord and nerve roots.

Postoperative imaging re-evaluation: Three-dimensional lumbar CT showed minimal bone removal from the superior margin of the right S1 lamina and the medial side of the inferior articular process of L5, with complete preservation of the bone at the inferior margin of the L5 lamina (Figure 4A); lumbar MRI and CT confirmed complete removal of the L5/S1 herniated nucleus pulposus, adequate decompression of the dural sac and S1 nerve root, a smooth posterior annular margin, no recurrence of disc herniation, and preservation of most of the ligamentum flavum (Figures 4B–E).

## 3 Discussion

### 3.1 Anatomical basis of the modified interlaminar approach

The “angular approach” initially proposed in this study is not an independent new surgical corridor anatomically, but a targeted optimization strategy of the traditional interlaminar approach. Therefore, it was revised to the modified interlaminar approach to ensure scientific and rigorous nomenclature. This approach takes the tip of the inferior articular process of L5 as the initial target. The surgical working window is located in the anatomical angular region formed by the inferior articular process of L5 and the superior margin of the S1 lamina, corresponding to the lumbar lateral recess. Compared with the traditional interlaminar approach targeting the spinolaminar junction, it has the following anatomical advantages: ① No need to resect the inferior margin of the upper vertebral lamina; only a small amount of bone from the superior margin of the S1 lamina and the medial part of the L5 inferior articular process is removed, maximally preserving the osseous structure of the spine. ② The working window directly corresponds to the region where L5/S1 disc herniation tends to compress the S1 nerve root, with stronger targeting and more direct decompression. ③ It avoids anatomical barriers in PTED for L5/S1 LDH, such as high iliac crest, hypertrophic L5 transverse process, and foraminal stenosis. Repeated puncture and angle adjustment are unnecessary, reducing radiation exposure and the risk of vascular injury (1, 2). The feasibility of this approach relies on the unique anatomical characteristics of the L5/S1 segment: the anterior-posterior diameter of the lateral recess in the L5/S1 interlaminar window reaches 8–12 mm, significantly wider than that of L3/4 and L4/5 segments (5–8 mm), providing sufficient operating space for a 4 mm endoscope and surgical instruments. Moreover, the tip of the L5 inferior articular process is a clear bony landmark, allowing accurate fluoroscopic localization and reducing technical difficulty.

### 3.2 Management strategy of the ligamentum flavum

The descriptions of “preserving most of the ligamentum flavum” and “resecting part of the ligamentum flavum” in this study are not contradictory, but represent refined management based on surgical requirements. Instead of blind resection, the ligamentum flavum is dissected along its longitudinal fibers, and only the local area sufficient to expose the herniated nucleus pulposus and nerve root is removed. The remaining ligamentum flavum is retracted medially with a nerve hook and preserved intact, avoiding excessive traction that may cause tearing. The advantages of this strategy are: ① Preserving most of the ligamentum flavum maintains the physiological barrier of the lumbar spinal canal, reduces epidural scar formation, lowers the risk of postoperative nerve root adhesion, and retains clear anatomical landmarks for potential revision surgery (3). ② It avoids injury to the posterior spinal column stability caused by extensive resection of the ligamentum flavum in the traditional interlaminar approach. ③ Local resection rather than full-thickness resection ensures sufficient surgical exposure and prevents incomplete nucleotomy due to inadequate working space. The principle adopted in this study is “retraction instead of resection as long as exposure is sufficient”, which also provides a practical reference for the management of the ligamentum flavum in spinal endoscopic surgery, balancing the needs of surgical exposure and spinal structure preservation.

### 3.3 Key points of annular suture technique

Annular rupture is a major cause of recurrent disc herniation. Previous studies reported that the recurrence rate after endoscopic surgery for lumbar disc herniation without annular repair is 6%–15%, while annular suture can significantly reduce the recurrence risk (4, 5). The technique used in this study—full-thickness annular suture + extra-canal knotting + knot-pushing with a dedicated knot pusher—addresses several limitations of conventional annular suturing. Its key technical points and advantages are: Full-thickness suture rather than partial suture: ensures tight closure of the annular defect, reduces the risk of re-herniation, and avoids poor healing caused by incomplete suturing. Extra-canal knotting: knotting is performed in the subcutaneous tissue with ample space, ensuring knot security and avoiding difficult knotting or loosening in the narrow spinal canal. Dedicated knot pusher: the knot is slowly pushed into place and attached to the annulus fibrosus, allowing precise control of tension and active adjustment of the knot position away from the nerve root, fundamentally avoiding mechanical irritation. 4-0 absorbable suture: the suture gradually degrades *in vivo*, avoiding inflammatory reactions from permanent foreign bodies, and provides appropriate tensile strength to meet mechanical demands during annular healing.

No patients in this study had postoperative nerve root irritation, and imaging re-examination showed a smooth posterior annulus, confirming the safety and effectiveness of this suture technique.

### 3.4 Objective comparison with traditional minimally invasive procedures

#### 3.4.1 Comparison between AUSS and UBE

With the rapid development of endoscopic techniques, single-channel endoscopic surgery (e.g., Arthroscopic-assisted Uni-portal Spine Surgery, AUSS) and dual-channel endoscopic surgery (e.g., Unilateral Biportal Endoscopy, UBE) have become important minimally invasive methods for lumbar degenerative diseases, with differences in technical concepts and procedures (6). Some scholars believe that UBE, with independent viewing and working channels, provides a “triangulation” effect similar to arthroscopy, offering flexible manipulation and a relatively gentle learning curve (7). Zhou S et al. noted that UBE involves more extensive soft-tissue dissection and trauma, whereas AUSS uses a single channel for both visualization and operation, theoretically causing less soft-tissue injury. A comparative study showed that AUSS was superior to UBE in operation time and incision length, with lower postoperative muscle injury (8). Compared with UBE, the single-portal spinal endoscopic modified interlaminar approach used in this study has the following advantages: ① Single incision (18 mm), limited paraspinal muscle dissection, mild soft-tissue injury, less postoperative pain, and faster recovery (9). ② Only a small amount of bone is removed, and most of the ligamentum flavum and facet joints are preserved, resulting in better spinal stability. ③ The single-portal non-coaxial design combined with the flexible viewing angle of a 30° arthroscope achieves surgical exposure and flexibility comparable to UBE, with simpler instrument manipulation and higher surgical efficiency.

Notably, UBE has more obvious advantages in treating L5/S1 LDH with severe spinal stenosis or massive disc herniation due to its larger working space. The present technique is more suitable for simple and mild-to-moderate L5/S1 LDH. The two techniques are complementary rather than mutually exclusive.

Surgeons with experience in conventional open or arthroscopic surgery may learn UBE more easily (7). The learning curve of AUSS is steeper; however, after overcoming the initial learning plateau, surgeons can achieve extremely high efficiency and accuracy. Evidence suggests that after proficiency, AUSS provides postoperative pain relief and quality of life similar to or even better than UBE (8).

#### 3.4.2 Comparison with traditional single-portal coaxial spinal endoscopy

Traditional single-portal coaxial spinal endoscopy has limitations such as a steep learning curve, low efficiency, limited visualization, narrow working space, and relatively high recurrence rate (10). The Arthroscopic-assisted Uni-portal Spine Surgery used in this study integrates the flexible manipulation of arthroscopy with the minimally invasive benefits of single-portal endoscopy: ① Non-coaxial design allows instruments and the endoscope to operate at an angle, avoiding instrument conflicts in coaxial systems and improving maneuverability. ② The 30° arthroscope enables multi-angle observation, clearly visualizing hidden regions in the spinal canal and reducing the risk of residual nucleus pulposus. ③ The high-resolution imaging system clearly displays microstructures such as microvessels and nerve endings, improving surgical precision and safety (11, 12).

### 3.5 Indications, contraindications, and learning curve

#### 3.5.1 Indications

① Isolated L5/S1 lumbar disc herniation with or without mild lumbar spinal stenosis, obvious nerve root compression, and ineffective conservative treatment. ② L5/S1 disc herniation with contraindications or limitations to PTED (e.g., high iliac crest, foraminal stenosis). ③ Young patients with L5/S1 LDH and high requirements for spinal stability. ④ Disc herniation with annular rupture and high risk of recurrence.

#### 3.5.2 Contraindications

① Massive L5/S1 disc herniation with severe lumbar spinal stenosis or spondylolisthesis. ② Spinal infection, tumor, or fracture at the L5/S1 segment. ③ Coagulopathy or inability to tolerate general anesthesia. ④ Severe dysfunction of the heart, lung, liver, kidney, or other major organs.

#### 3.5.3 Learning curve

The learning curve of this technique is relatively gentle. Based on conventional interlaminar endoscopy, surgeons can master it quickly by grasping three key technical points: ① Accurate fluoroscopic localization of the tip of the L5 inferior articular process. ② Refined management of the ligamentum flavum under the principle of “retraction instead of resection as long as exposure is sufficient”. ③ The procedure of full-thickness annular suture + extra-canal knotting + knot pushing.

For surgeons with basic spinal endoscopic experience, proficiency can be achieved after 3–5 cases.

### 3.6 Study limitations

This study is a single-case report with a small sample size and only 12 months of follow-up. Therefore, long-term efficacy, disc recurrence rate, and long-term effects on spinal stability cannot be determined. No control group was included, so head-to-head comparison with traditional procedures was not performed. Annular healing was only indirectly evaluated by imaging without histological verification.

Future studies should include large-sample, multi-center, prospective controlled designs with longer follow-up to further verify long-term safety and effectiveness. Biomechanical studies can be conducted to evaluate the effects on L5/S1 segment biomechanics. Additionally, technical optimization and feasibility in upper lumbar segments such as L4/5 can be explored.

## 4 Conclusion

Arthroscopic-assisted Uni-portal Spine Surgery via the modified interlaminar approach combined with full-thickness annular suture is a safe, effective, and minimally invasive procedure for L5/S1 lumbar disc herniation. By targeted optimization of the traditional interlaminar approach, this technique avoids anatomical barriers and maximally preserves the osseous and ligamentous structures of the spine. The full-thickness annular suture with extra-canal knotting achieves secure annular repair, reduces the risk of postoperative recurrence, and avoids knot irritation to the nerve root. This technique is easy to perform and highly efficient, providing a new therapeutic option for L5/S1 disc herniation, especially cases limited by conventional endoscopic procedures. It has promising clinical application prospects, but further validation through large-sample and long-term follow-up studies is required.

## Data Availability

The raw data supporting the conclusions of this article will be made available by the authors, without undue reservation.
